# Leptin induces muscle wasting in a zebrafish *kras*-driven hepatocellular carcinoma (HCC) model

**DOI:** 10.1242/dmm.038240

**Published:** 2019-02-27

**Authors:** Qiqi Yang, Chuan Yan, Xu Wang, Zhiyuan Gong

**Affiliations:** 1 Department of Biological Sciences, National University of Singapore, 117558, Singapore; 2 Key Laboratory of Metabolism and Molecular Medicine, Ministry of Education, Department of Biochemistry and Molecular Biology, School of Basic Medical Sciences, Fudan University, Shanghai 230002, China

**Keywords:** Leptin, Leptin receptor, Lepr, Muscle wasting, Cancer cachexia, Liver cancer, Zebrafish

## Abstract

Cancer cachexia affects up to 80% of patients with advanced solid cancer and leads to excessive muscle wasting. Here, using an inducible zebrafish hepatocellular carcinoma (HCC) model driven by oncogenic *kras^G12V^*, we observed a progressive muscle-wasting phenotype in adult zebrafish, characterized by significant loss of body weight and muscle fibers. By differential feeding, we observed that overfeeding caused fatty liver, accelerated carcinogenesis and muscle wasting. Interestingly, leptin, an obesity hormone, was upregulated in oncogenic hepatocytes and overfeeding groups. We also found that leptin expression progressively increased during human liver disease progression. By using leptin receptor (*lepr*)-knockout fish, we found that tumor fish in the *lepr* mutant background had a higher survival rate and significantly lower muscle-wasting level after tumor induction than the tumor fish in the wild-type background. Chemical inhibitors targeting leptin signaling also alleviated the muscle-wasting phenotype, indicating that leptin signaling may be a new therapeutic target for cancer patients with muscle wasting.

## Abstract (precis)

**Summary:** Through a zebrafish model, this study demonstrates that leptin plays an important role in cancer-induced muscle wasting and that the leptin pathway may be a therapeutic target in cancer cachexia.

## INTRODUCTION

Cancer cachexia is a devastating syndrome that affects up to 80% of human patients with advanced cancer and may account for up to 20% of cancer deaths (Argiles et al., 2014; Teunissen et al., 2007). The occurrence of cachexia is often accompanied by substantial loss of body weight (body mass) caused by wasting of skeletal muscle (Lok, 2015), which is considered to be a crucial hallmark of cancer cachexia (Ballaro et al., 2016). Even though skeletal muscle is the major tissue affected by cachexia, muscle wasting and cancer cachexia are not equivalent syndromes (Porporato, 2016). Several other organs, such as fat tissue, heart, gut and brain, could be affected, making cachexia a multi-organ syndrome (Argiles et al., 2014). In hepatocellular carcinoma (HCC) patients undergoing curative treatments, muscle wasting occurs in 40% of patients and is predictive of low survival (Harimoto et al., 2013). The underlying molecular mechanism remains largely unknown and there is no specific drug for treatments of human patients with muscle wasting (Winbanks et al., 2016).

Muscle wasting has been studied in mouse and rat models by quantification of loss of gross body weight. Injection of human kidney cancer cells induced 30% of body weight loss in mice after 40 days and led to a high level of p38 stress-response signature in skeletal muscle (Fukawa et al., 2016). Also, injection of the carcinogen diethylnitrosamine induced irreversible hepatocellular carcinogenesis and subsequently 50% of injected mice displayed a significant loss of body weight after 19 months (Park et al., 2009; Petruzzelli et al., 2014). As cancer-associated muscle wasting has not been previously reported in zebrafish tumor models, here our first aim is to characterize loss of gross body weight and muscle fiber in our *kras^G12V^*-induced HCC model (Chew et al., 2014) and prove it to be a useful muscle-wasting model.

In order to study whether loss of body weight and muscle fibers could be mitigated by dietary therapies, nutrient supplementation on weight-loss patients has been investigated, but these studies have yielded controversial results (Gullett et al., 2011). In a retrospective study of recorded weight change and changes in dietary intake of patients with advanced cancer over 6 years, there is no overall correlation between increase of dietary intake and body weight, contrary to what was expected. In a meta-analysis of 13 studies on oral nutritional interventions on malnourished patients (mainly patients with cancer or chronic diseases), there is no statistically significant improvement of mortality in nutritional intervention groups compared to control groups (Baldwin et al., 2012). Meanwhile, starvation therapy has been proposed as a cancer treatment and may help improve cancer immunotherapy or chemotherapy (Safdie et al., 2009; Zhong et al., 2016). In our zebrafish HCC-induced muscle-wasting model, our second aim is to investigate the effects of nutrients on carcinogenesis and muscle wasting.

Molecularly, we found that leptin, an obesity hormone, showed a significant upregulation after *kras^G12V^* induction. Leptin is an adipostatic hormone expressed by adipocytes to regulate adipose mass in mammals (Halaas et al., 1995), and inhibition of leptin signaling leads to obesity or diabetes (Clement et al., 1998). However, in fish, leptin is predominantly expressed in the liver (Denver et al., 2011; Michel et al., 2016). Although knockout of the leptin receptor (*lepr*) gene failed to cause body obesity in zebrafish, a previous study showed that the adipostatic role of leptin in the regulation of β-cell number, insulin expression and glucose homeostasis remained conserved across vertebrates (Michel et al., 2016). In this study, our third aim is to identify whether leptin signal transduction is associated with muscle wasting. Also, we would like to identify whether chemical inhibition of the main downstream components of leptin signaling had a significant effect on the treatment of muscle wasting.

## RESULTS

### Characterization of zebrafish muscle wasting after HCC induction

Previously, we have developed an inducible *kras* transgenic zebrafish model using the tetracycline-controlled transcription activation (Tet-On) inducible system, in which reverse tetracycline-controlled transactivator (rtTA) protein is capable of binding to tetracycline response element (TRE) only if bound by tetracycline or an analog, doxycycline (dox). By using this Tet-On system, rtTA is expressed under the hepatocyte-specific *fabp10* promoter and the effector fusion gene, *kras^G12V^-EGFP*, under TRE (Fig. S1A). Hence, in the transgenic model, expression of *kras^G12V^-EGFP* in hepatocytes is induced by extraneous introduction of dox for liver tumorigenesis (Chew et al., 2014).

**Fig. 1.**
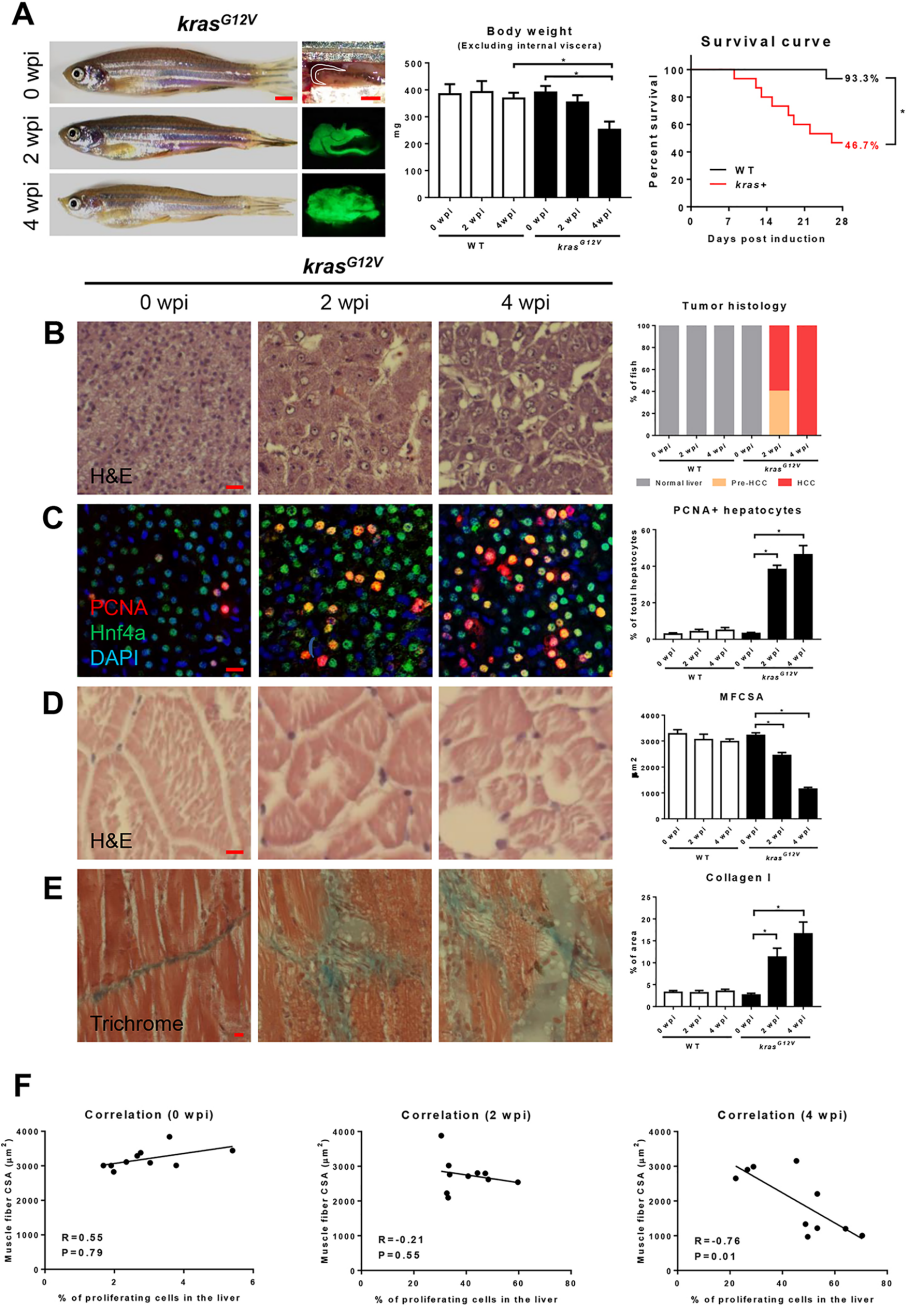
**Characterization of zebrafish muscle wasting in *kras^G12V^*-induced HCC zebrafish.** 4-month-old male adult *kras^G12V^* and WT zebrafish were treated with dox for 4 weeks and sampled at 0 wpi, 2 wpi and 4 wpi. In each group, 15 fish were used to initiate the experiments. (A) Gross appearance and liver morphology (left), body weight excluding internal viscera (middle) and survival curves (right). (B) H&E staining of liver sections of *kras^G12V^* fish. Quantification of tumor histology (right). (C) IF staining of PCNA (red), Hnf4a (green) and DAPI (blue) in liver sections of *kras^G12V^* fish. Quantification of percentage of proliferating hepatocytes (right). (D) H&E staining of muscle sections of *kras^G12V^* fish. Quantification of MFCSA (right). (E) Gomori's trichrome staining of muscle sections of *kras^G12V^* fish. Quantification of percentage of collagen deposited area (right). (F) Correlation between percentage of proliferating cells in the liver (*x*-axis) and MFCSA (*y*-axis) at 0 wpi (left), 2 wpi (middle) and 4 wpi (right). *P<0.05. Scale bars: 2.5 mm in A; 10 μm in B-E.

To characterize HCC-induced muscle wasting, 4-month-old male wild-type (WT) and *kras^G12V^* fish were exposed to dox for 4 weeks. Samples were collected at 2 weeks post-induction (wpi) and 4 wpi. Gross morphology and liver morphology showed that *kras^G12V^*-expressing livers had a significant liver enlargement at 2 wpi and were further enlarged at 4 wpi. After 4 weeks of induction, tumor-bearing fish looked thinner and leaner (Fig. 1A, left panel). Body weight excluding internal viscera was reduced significantly in *kras^G12V^* fish at 4 wpi compared to the WT control siblings (Fig. 1A, middle panel). Only 46.7% of *kras^G12V^* fish survived the treatments (Fig. 1A, right panel). Most *kras^G12V^* fish died owing to advanced tumor progression (data not shown). Histologically, *kras^G12V^* fish at 0 wpi had typically normal liver histology, with hepatocytes arranged into regular two-cell-thick plates as described for human liver histology (Gissen and Arias, 2015). At 2 wpi, 60% of *kras^G12V^* fish developed HCC characterized by the total abrogation of the two-cell plate, appearance of prominent nucleoli, hyperchromatism, irregular nuclear borders and hepatic vacuolation. At 4 wpi, all the *kras^G12V^* fish developed HCC with more pleomorphism, nuclear irregularity and angulated nuclei, indicating the more advanced and late HCC stage (Fig. 1B). Furthermore, we observed a significantly higher rate of hepatocyte proliferation in *kras^G12V^* fish at 2 wpi, which was further increased at 4 wpi (Fig. 1C). Histological analyses revealed that *kras^G12V^* fish sustained severe skeletal muscle wasting with a progressively reduced muscle fiber cross-sectional area (MFCSA) (Fig. 1D), which is commonly used to indicate muscle fiber size (Fukawa et al., 2016). Fibrosis progression is assumed as a secondary phenomenon in muscle wasting and has been proposed as a compensatory replacement of lost muscle (Klingler et al., 2012). Here, we observed an increased level of fibrosis along with the loss of muscle fibers (Fig. 1E). Interestingly, we found that, during carcinogenesis, MFCSA showed a significantly negative correlation with percentage of proliferating hepatocytes at 4 wpi, indicating that only the advanced tumors were associated with severe muscle wasting (Fig. 1F, right panel). Results on WT fish are presented in Fig. S2 and there was no significant difference during the 4 weeks of dox induction. Hence, we identified a useful muscle-wasting model in the *kras^G12V^*-induced-HCC zebrafish.

We also observed reduced muscle wasting in female fish after *kras^G12V^* induction under the same conditions (data not shown), apparently because of the slower tumor progression in females compared with males (Li et al., 2017; Yan et al., 2017). To avoid the gender effect, only male fish were used in the subsequent experiments.

### Increased food supplementation accelerated hepatocarcinogenesis and muscle wasting

**Fig. 2.**
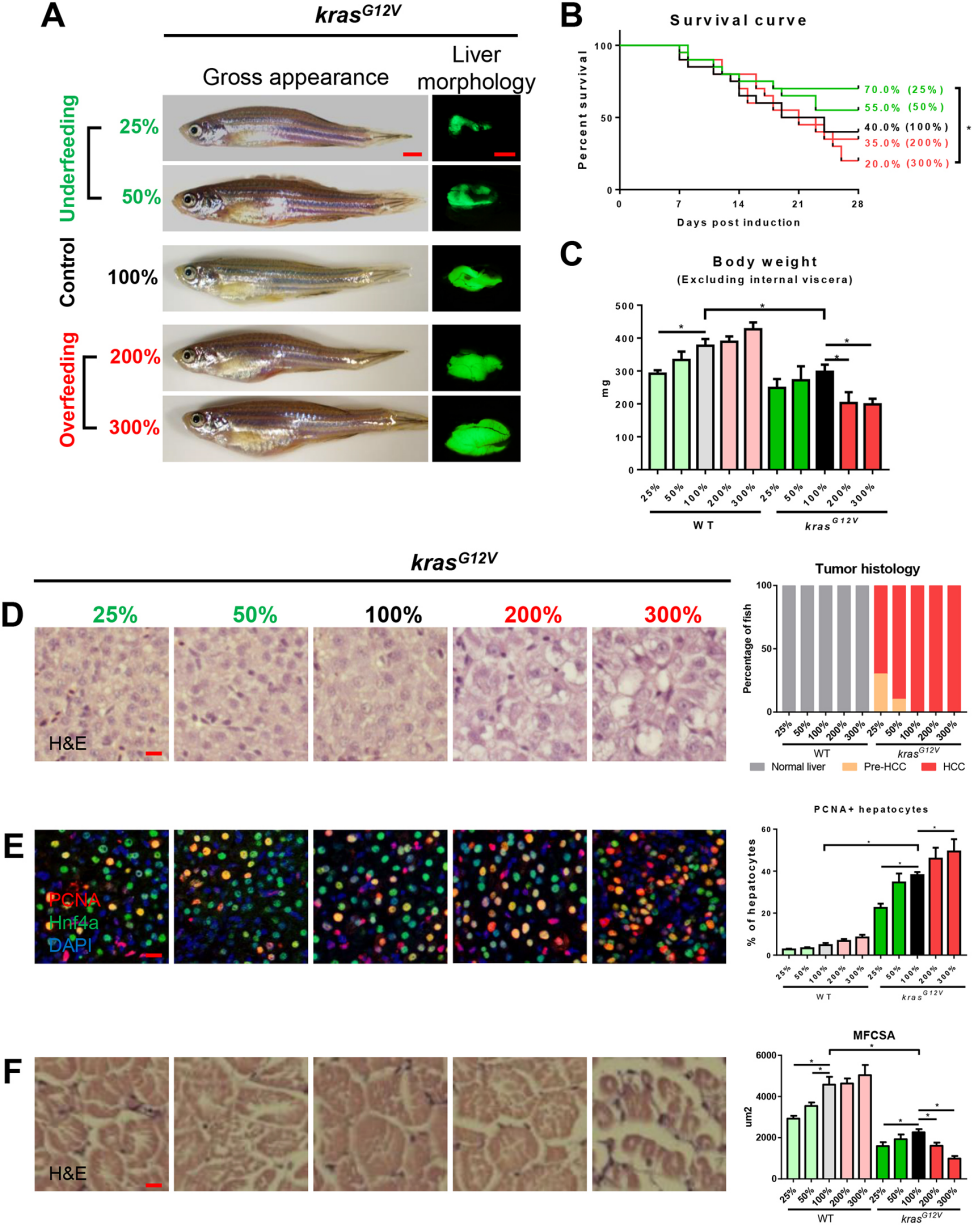
**Effects of manipulation of food supplementation on hepatocarcinogensis and muscle wasting.** 4-month-old male adult *kras^G12V^* and WT zebrafish were treated with dox for 4 weeks and fed with different levels of artemia. A total of 5 mg artemia cysts/fish/day for normal feeding was given, defined as 100%. There were two underfeeding groups with 25% and 50% the amount of food of normal feeding, and two overfeeding groups with 200% and 300% the amount of normal feeding. In each feeding group, 20 fish were used to initiate the experiments. (A) Gross appearance and liver morphology based on liver-specific GFP expression in *kras^G12V^* fish. (B) Survival curves. (C) Body weight excluding internal viscera. (D) H&E staining of liver sections of *kras^G12V^* fish (left) and quantification (right). (E) IF staining of PCNA (red), Hnf4a (green) and DAPI (blue) in liver sections. Quantification of percentage of proliferating hepatocytes (right). (F) H&E staining of muscle sections of *kras^G12V^* fish. Quantification of MFCSA of *kras^G12V^* fish (right). *P<0.05. Scale bars: 2.5 mm in A; 10 μm in D-F.

To investigate the effects of nutrients on carcinogenesis and muscle wasting, 4-month-old zebrafish were fed with different doses of artemia for 4 weeks after *kras^G12V^* induction. A diet of 5 mg artemia cysts/fish/day was used as normal feeding, defined as 100%. Then, we designed two underfeeding groups, with 25 and 50% of normal feeding, to test the effects of starvation, and two overfeeding groups, with 200% and 300% of normal feeding, to determine the effects of excess nutrients. Morphologically, in *kras^G12V^* fish, we observed fatty bodies in both overfeeding groups and relatively thin bodies in the underfeeding and normal feeding groups. The liver size was progressively increased with increasing feeding (Fig. 2A). Reduction of food intake improved the overall survival from tumor burden (Fig. 2B). Although both underfeeding and overfeeding groups had no significant difference compared to the normal feeding group, there was a significant improvement of survival in the 25% feeding group compared to the 300% feeding group, indicating that overfeeding accelerated tumorigenesis. As shown in Fig. 2C, WT fish had a consistent increase in body weight with increased food intake, while, in *kras^G12V^* fish, overfeeding has an unexpected reduction of body weight compared with normal feeding. Histologically, we observed that 100% of *kras^G12V^* fish in the normal and overfeeding groups developed HCC, while underfeeding attenuated HCC progression, with 70 and 90% of *kras^G12V^* fish developing HCC in the 25 and 50% feeding groups, respectively (Fig. 2D). Furthermore, nutrient administration accelerated tumor cell proliferation after *kras^G12V^* induction (Fig. 2E). Consistent with the changes of body weight, MFCSA was reduced significantly after *kras^G12V^* induction, and showed further decreases in both the underfeeding and overfeeding groups (Fig. 2F, Fig. S3E). All the WT fish data are shown in Fig. S3; there were no significant differences between different feeding groups.

Our data indicated that extra nutrient administration promoted both tumor progression and muscle wasting. Underfeeding, though, attenuated carcinogenesis and also accelerated muscle wasting, likely due to the reduced food intake. Interestingly, the overfeeding groups showed stronger muscle wasting than the underfeeding groups, indicating that tumor-associated factors play a larger role in muscle wasting.

### Fatty-liver-associated upregulation of leptin after *kras^G12V^* induction in zebrafish and in human liver disease patients

**Fig. 3.**
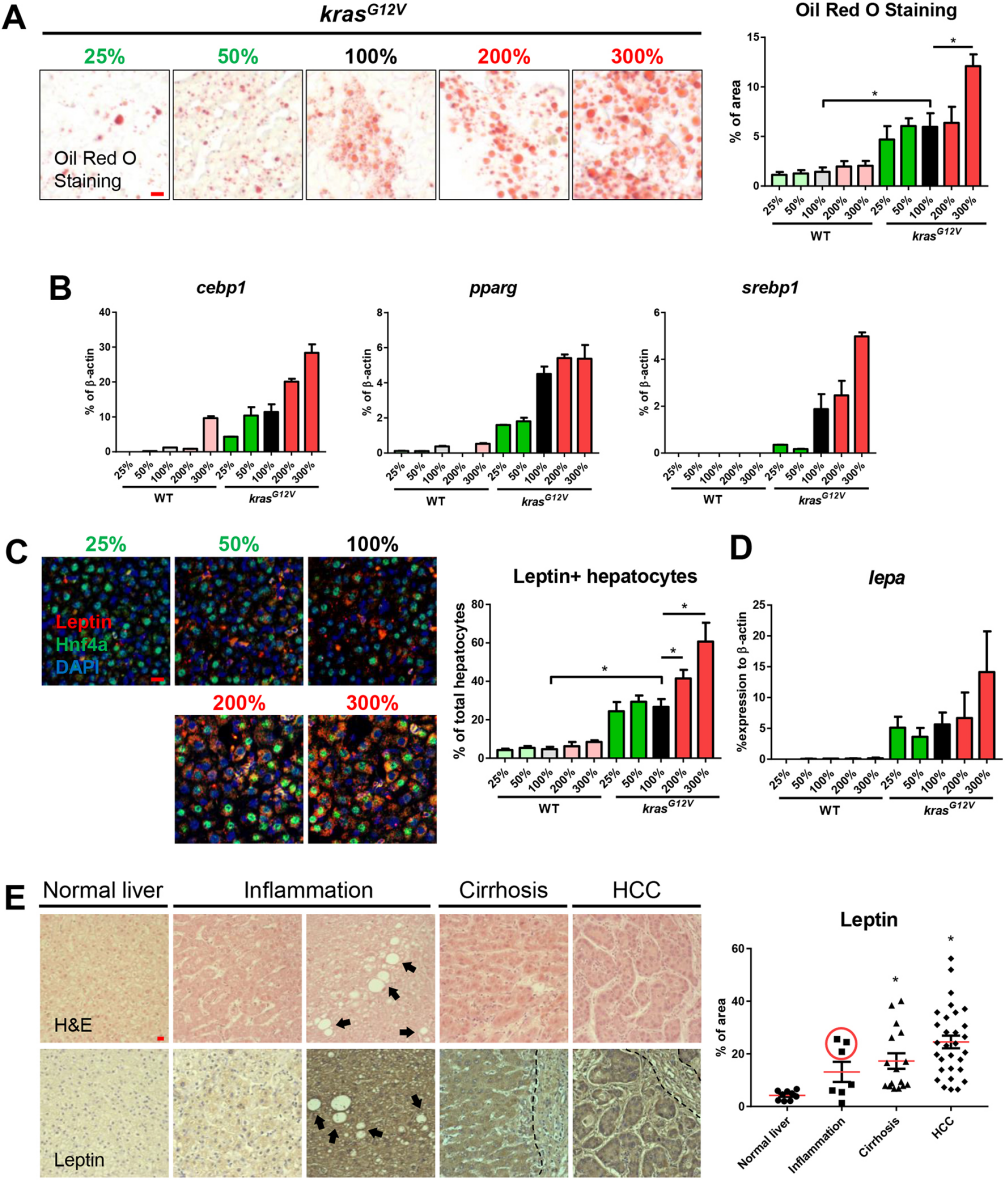
**Fatty-liver-associated upregulation of leptin in zebrafish after *kras^G12V^* induction and in human liver disease patients.** 4-month-old male adult *kras^G12V^* and WT zebrafish were treated with dox for 4 weeks and fed with different levels of artemia. (A) Oil Red O stain of liver sections of *kras^G12V^* fish (left) and quantification of percentage area with accumulated fat (right). (B) Expression of lipogenic genes (*cebp1*, *pparg* and *srebp1*) in livers of *kras^G12V^* and WT fish. (C) IF staining of leptin (red), Hnf4a (green) and DAPI (blue). Quantification of percentage of leptin-expressing hepatocytes (right). (D) Expression of *lepa* in livers of *kras^G12V^* and WT fish. (E) H&E staining (top row) and leptin expression (bottom row) of human liver disease samples. Patient samples were characterized into normal liver, inflammation, cirrhosis and HCC. Both low (left) and high (right) inflammation samples are shown and arrows indicate the presence of possible lipid droplets. The dashed line delimits the boundary of blood vessel. Quantification of leptin expression level in patient samples (right). The three high-leptin-expressing samples are circled in the inflammation group. *P<0.05. Scale bars: 10 μm.

Fatty liver is one of the major risk factors in HCC initiation and progression (Severi et al., 2010). By Oil Red O staining, we found a significant increase of lipid droplets in *kras^G12V^* fish in the overfeeding groups (Fig. 3A); in comparison, WT fish showed little change in Oil Red O staining in different feeding groups (Fig. S4A). Expression of the lipogenic genes *cebpa*, *pparg* and *srebp1* was also significantly upregulated after *kras^G12V^* induction, and was further increased by overfeeding (Fig. 3B).

Leptin is the primary adipostatic hormone to regulate food intake and energy balance in humans (Halaas et al., 1995). In zebrafish, leptin is mainly expressed in hepatocytes (Denver et al., 2011; Michel et al., 2016). By immunofluorescence co-staining of leptin and Hnf4a (Hepatocyte nuclear factor 4a), the hepatocyte marker, we found a significant increase of leptin after *kras^G12V^* induction in oncogenic hepatocytes, and the increase was further enhanced by overfeeding (Fig. 3C, Fig. S4B). Reverse-transcription quantitative polymerase chain reaction (RT-qPCR) showed a consistent upregulation of *lepa* in *kras^G12V^* fish and in the overfeeding groups (Fig. 3D), while *lepb* expression was barely detectable in both normal and tumor livers (data not shown). Thus, there was a positive association of overfeeding with both fatty-liver progression and leptin expression.

It has been reported that serum leptin level is increased significantly in human patients with liver cirrhosis and HCC (Wang and Lin, 2003). To investigate leptin expression during liver disease progression, a panel of human liver disease samples were examined. These samples included normal liver (*n*=9), inflammation (*n*=7), cirrhosis (*n*=16) and HCC (*n*=30). Hematoxylin and eosin (H&E) and immunohistochemistry (IHC) staining for human leptin were conducted. We found significant upregulation of leptin in cirrhosis and HCC samples (Fig. 3E). However, inflammation samples were clearly separated into two groups, low leptin expression (*n*=4) and high leptin expression (*n*=3) (Fig. 3E). Interestingly, all of the three high leptin expression samples had fatty-liver characteristics, with round and empty vesicles (indicated by arrows in Fig. 3E), which are apparently the lipid droplets (Levene et al., 2012). Thus, there was also a potential correlation between leptin expression and fatty liver progression in human samples, and it seems that leptin signaling is upregulated throughout the disease progression from pro-HCC to HCC.

### Knockout of leptin receptor gene (*lepr*) attenuated *kras^G12V^*-induced muscle wasting

**Fig. 4.**
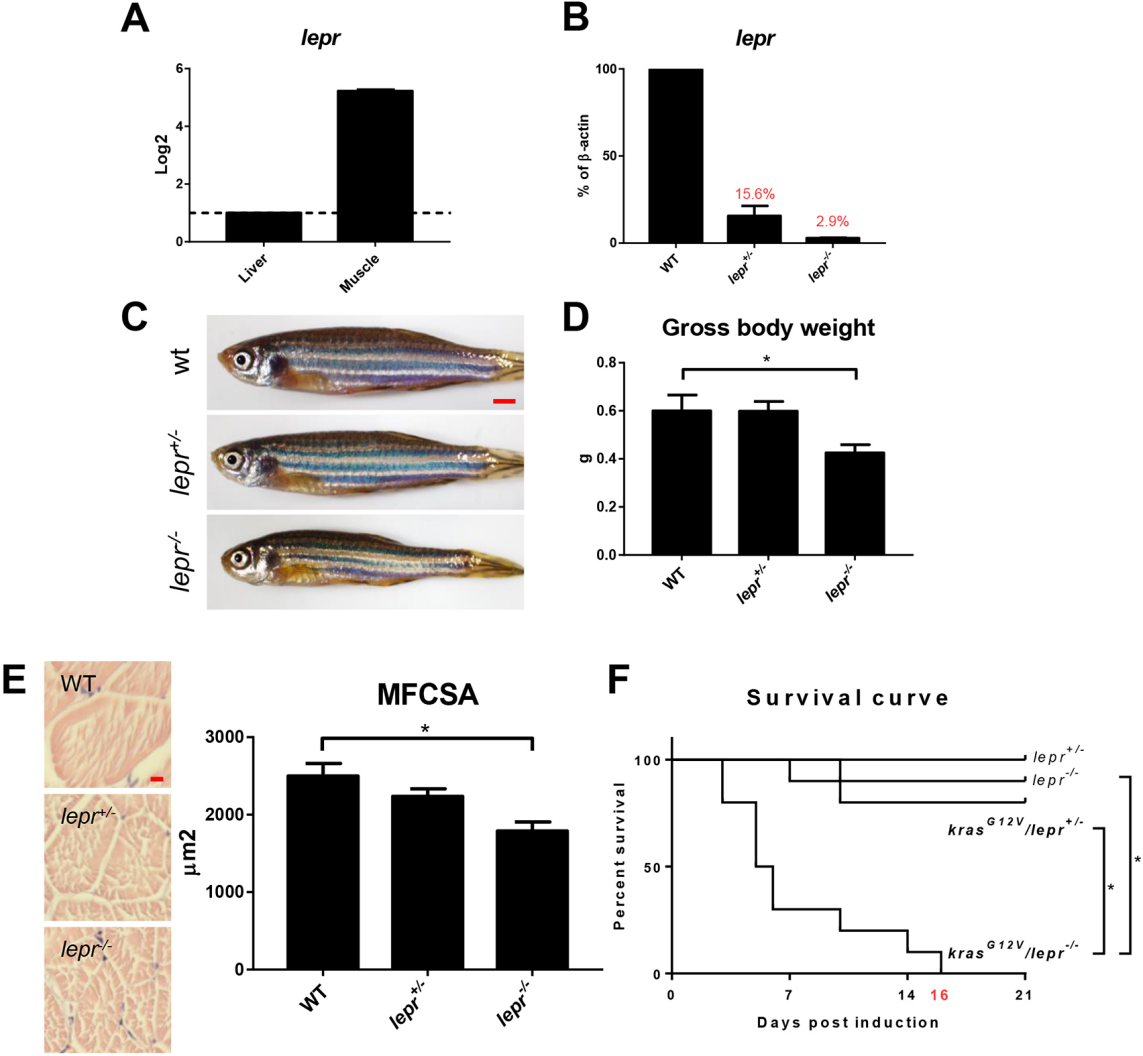
**Expression of *lepr* and characterization of *lepr* mutant zebrafish.** (A) RT-qPCR analyses of relative *lepr* mRNA expression in the liver and skeletal muscle in WT fish. The value of liver expression is arbitrarily set as 1. (B) RT-qPCR analyses of *lepr* mRNA expression in skeletal muscles in WT, *lepr^+/−^* and *lepr^−/−^* mutant zebrafish. Relative expression levels were the values with WT muscles set as 1. (C) Gross appearance of 4-month-old WT, heterogeneous and homogenous *lepr* mutant zebrafish. (D) Gross body weight. (E) H&E staining of muscle sections and quantification of MFCSA. (F) Survival curves after *kras^G12V^* induction in heterogeneous mutant fish, homogenous mutant fish and non-*kras^G12V^* controls. In each group, 10 fish were used to initiate the experiments. *P<0.05. Scale bars: 2.5 mm in C; 10 μm in E.

Both *kras^G12V^* induction and overfeeding upregulated leptin expression (Fig. 3C,D), indicating a potential contribution of leptin in muscle wasting. To establish the role of leptin in muscle wasting, the level of expression of the leptin receptor gene, *lepr*, was measured in muscle by RT-qPCR. Indeed, *lepr* had a much higher expression in skeletal muscle than in liver (Fig. 4A). To investigate whether the tumor- and overfeeding-induced muscle wasting was related to leptin signal, the single *lepr* gene in the zebrafish genome (Liu et al., 2010) was knocked out by CRISPR (Fei et al., 2017). The mutation resulted in a truncated Lepr protein with predicted null function (Fei et al., 2017). Indeed, we found significant loss of *lepr* mRNAs in skeletal muscles in both heterozygous and homozygous *lepr* mutants (Fig. 3B). Gross morphology after 4 months post-fertilization (mpf) showed that the homozygous mutant fish had a thinner body than heterozygous mutant and WT fish (Fig. 4C). Homozygous mutant fish showed a significant decrease of body weight (Fig. 4D) and muscle fiber size, compared with heterozygous and WT fish (Fig. 4E). Then, we induced *kras^G12V^* expression in *lepr* mutant fish, but all the *kras^G12V^/lepr^−/−^* fish died after 16 days of *kras^G12V^* induction (Fig. 4F). However, *kras^G12V^* induction in the WT background showed no significant loss of body weight after 2 wpi. Hence, *lepr*^+/−^ fish were used for *kras^G12V^* induction in the following experiments.

**Fig. 5.**
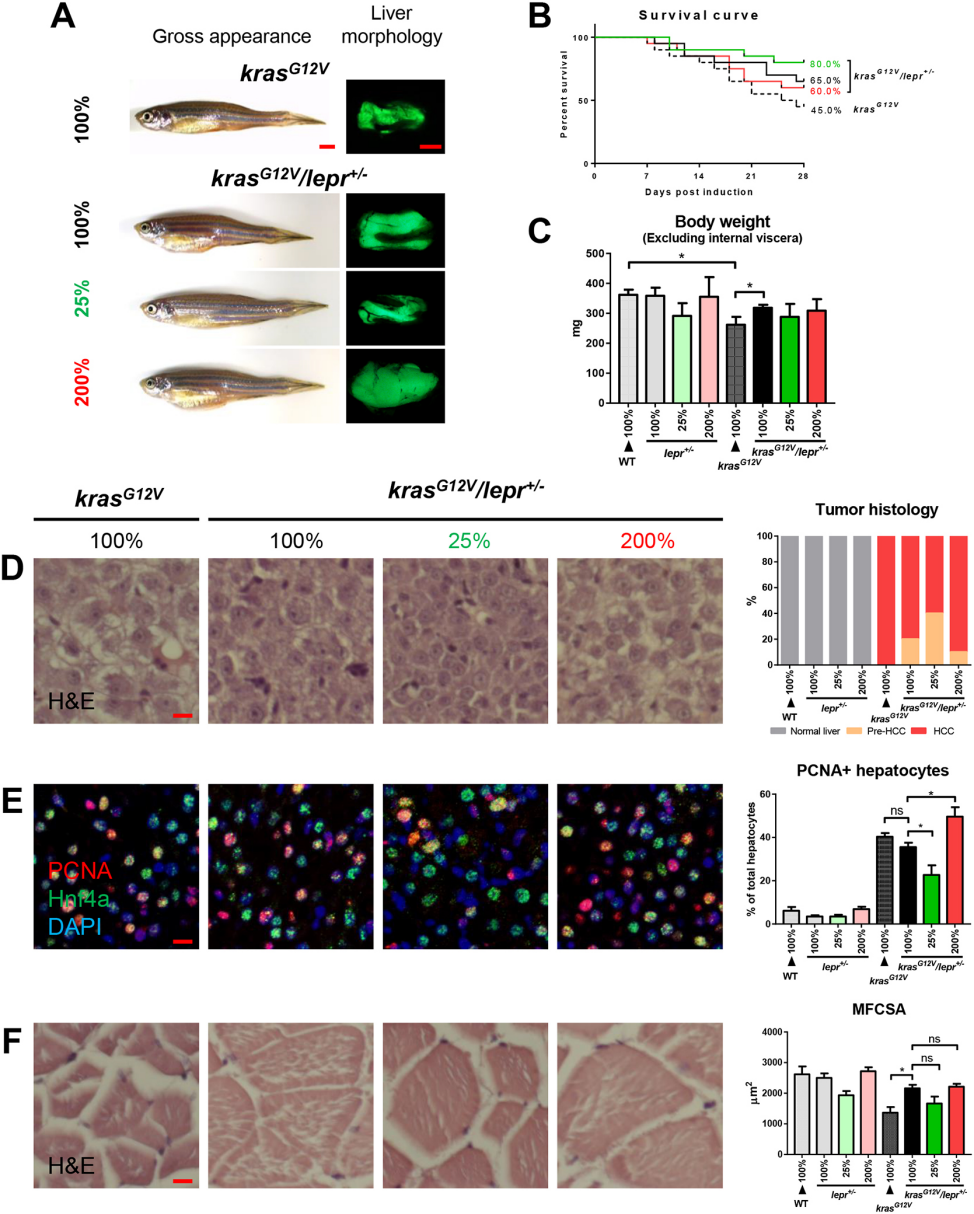
***kras^G12V^* induction in *lepr* heterogeneous mutant fish under different levels of feeding.** *lepr*^+/−^ fish with and without *kras^G12V^* were fed with 25%, 100% and 200% of artemia. Non-mutant WT and *kras^G12V^* fish were fed with 100% of artemia as controls for 4 weeks. In each feeding group, 20 fish were used to initiate the experiments. (A) Gross appearance and liver morphology based on liver-specific GFP expression in *kras^G12V^* fish. (B) Survival curves. (C) Body weight excluding internal viscera. (D) H&E staining of liver sections (left) and quantification (right). (E) IF staining of PCNA (red), Hnf4a (green) and DAPI (blue). Quantification of percentages of proliferating hepatocytes is presented on the right. (F) H&E staining of muscle sections (left) and quantification of MFCSA (right). *P<0.05. Scale bars: 2.5 mm in A; 10 μm in D-F.

To test whether *lepr* mutation could attenuate tumor- and overfeeding-induced muscle wasting, the *kras^G12V^/lepr^+/−^* fish were fed with 25, 100 and 200% of artemia. WT and *kras^G12V^* fish were fed with 100% of artemia as control (Fig. 5A). After 4 weeks of *kras^G12V^* induction, the *kras^G12V^/lepr^+/−^* fish showed an improved trend of survival rate in all three feeding groups, compared to *kras^G12V^* fish in the WT background (Fig. 5B). In comparison, survival rates were similar in all non*-kras^G12V^* fish groups (Fig. S5B). The *kras^G12V^* fish suffered a significant body weight loss that was alleviated in the *kras^G12V^/lepr^+/−^* fish (Fig. 5C). Similar to the observation in *kras^G12V^* fish (Fig. 2), underfeeding attenuated carcinogenesis, while overfeeding accelerated it in *kras^G12V^/lepr^+/−^* fish based on histological analyses and PCNA staining (Fig. 5D,E). Furthermore, the *lepr^+/−^* fish greatly reduced *kras^G12V^*-induced muscle wasting (Fig. 5F). Interestingly, overfeeding in *lepr^+/−^* fish led to comparable body weights (data not shown) and muscle fiber sizes to the normal and underfeeding groups (Fig. S5E), indicating that blocking leptin signaling also prevented overfeeding-induced further loss of body weight and muscle fibers.

Our findings indicated that knockout of *lepr*, although it had no effects on carcinogenesis, prevented both tumor- and overfeeding-induced muscle wasting. In comparison, knockout of *lepr* showed no effects on the liver and muscle fibers in non-*kras^G12V^* fish groups (Fig. S5).

### Targeting leptin signaling for muscle-wasting therapy

**Fig. 6.**
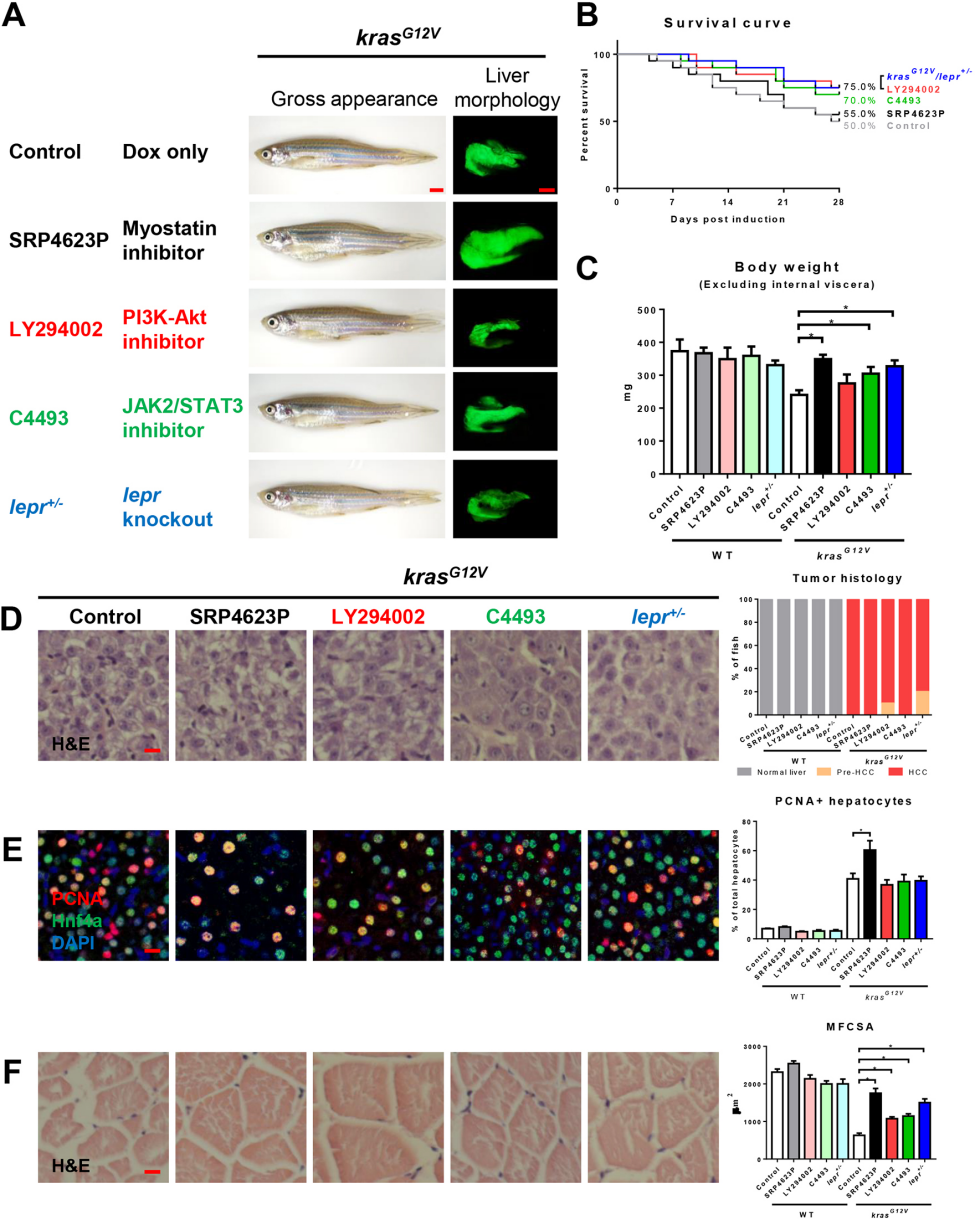
**Effects on hepatocarcinogenesis and muscle wasting of chemical inhibitors targeting downstream of leptin in the signaling pathway.** 4-month-old adult male *kras^G12V^* zebrafish were treated with dox for 4 weeks with or without SRP4623, LY294002 or C4493 treatment, in comparison with similarly dox-induced *kras^G12V^/lepr^+/−^* fish. In each group, 20 fish were used to initiate the experiments. (A) Gross appearance and liver morphology (left lateral view). Information of targets of chemical inhibitors is shown. (B) Survival curves. (C) Body weight excluding internal viscera. (D) H&E staining of liver sections (left) and quantification of tumor histology (right). (E) IF staining of PCNA (red), Hnf4a (green) and DAPI (blue). Quantification of percentage of proliferating hepatocytes is presented on the right. (F) H&E staining of muscle sections. Quantification of MFCSA is shown on the right. *P<0.05. Scale bars: 2.5 mm in A; 10 μm in D-F.

The main downstream signaling pathways of leptin are PI3K/AKT and JAK2/STAT3 (Zhou and Rui, 2013). Hence, we would like to identify the effects of chemical inhibitors of leptin downstream signaling on muscle. LY294002 and C4493 are inhibitors of PI3K/AKT and JAK2/STAT3, respectively (Jiang et al., 2010; Lim et al., 2006). In order to test whether leptin signaling could be a therapeutic target for treatment of tumor-induced muscle wasting, these two chemical inhibitors were employed. In clinical therapy for muscle wasting in cachexia patients, myostatin inhibitors have been widely investigated for therapeutic treatments (Gallot et al., 2014; Zhou et al., 2010); thus, the myostatin inhibitor SRP4623P was also used as a positive control to prevent muscle wasting (Smith and Lin, 2013). LY294002 and C4493 up to 20 µM were tested in *kras^G12V^* larvae and had no significant effects on carcinogenesis (data not shown). *kras^G12V^* fish in both WT and *lepr^+/−^* backgrounds were treated with dox and one of these chemical inhibitors at the highest all-survival concentration (10 µM) for 4 weeks. Gross morphology showed no significant differences among different treatments (Fig. 6A). However, SRP4623P, LY294002 or C4493 treatments plus *lepr* knockout resulted in an increased trend of survival rate compared to *kras^G12V^* fish after 4 weeks of dox induction (Fig. 6B). Myostatin inhibitor plus *lepr* knockout significantly prevented the loss of body weight (Fig. 6C). Liver histology and PCNA+ hepatocytes showed that all the treatments had no significant effects on carcinogenesis except that SRP4623P appeared to cause a significant increase of hepatocyte proliferation (Fig. 6D,E). Histologically, MFCSA was increased dramatically after myostatin inhibitor treatment. LY294002 or C4493 treatments plus *lepr* knockout also alleviated tumor-induced loss of muscle fibers (Fig. 6F).

Here, our findings show that both blockage of leptin downstream signaling and application of a myostatin inhibitor showed improvements in treatment of muscle wasting. All the data on treatments with WT fish are shown in Fig. S6 and no significant difference was observed among different chemical treatments.

## DISCUSSION

### A muscle-wasting model in liver-tumor transgenic zebrafish

Recently, cancer cachexia has been investigated in many experimental models and human patients. Many studies have suggested potential molecular mechanisms for clinical therapy (Ballaro et al., 2016). As a complex metabolic syndrome, cachexia is characterized by multiple factors, such as loss of muscle or loss of fat mass, heart failure, systemic inflammation, or anorexia (Konishi et al., 2015). Loss of adipose mass and decrease of fat cell volume have been widely characterized, and genes related to energy turnover are upregulated in cachexia patients (Dahlman et al., 2010). In a study of 177 individual patients who died of cancer, researchers found that 30.5% of patients with lung, pancreatic or gastrointestinal cancer had severe heart failure and significant loss of heart weight (Barkhudaryan et al., 2017). The effects on multiple organs could be due to the systemic inflammation and immune cell infiltration into various tissues. Comparison between the weight-loss and weight-stable patients under the same tumor diagnosis demonstrated the significant activation of macrophages and upregulation of inflammatory cytokines in cachexia patients, indicating the stimulating role of inflammation in cachexia patients (de Matos-Neto et al., 2015). Furthermore, anorexia is commonly present in cancer cachexia patients and has various causes (Ezeoke and Morley, 2015).

Among all these affected tissues and organs, wasting of skeletal muscle has been regarded as the most severe and significant syndrome in human patients with cancer cachexia. Hence, in our current study, we aimed to generate a muscle-wasting model in an inducible HCC transgenic zebrafish. In some previous animal models, tumors were produced by transplanting human tumor cells or by administering various carcinogens; hence, the level of cancer cachexia varied depending on cancer cell type, cell number and injection site (Konishi et al., 2015). Our genetically engineered models could provide more stable and more easily controlled tumor development and muscle-wasting phenotype. By using an inducible system to specifically express *kras^G12V^* in hepatocytes, HCC could be developed after 1 week of induction (Chew et al., 2014). After tumor induction, subsequent tumor-induced loss of body weight and muscle wasting became apparent within 4 weeks of tumor induction, which is faster than the majority of mouse muscle-wasting models (Park et al., 2009; Petruzzelli et al., 2014). Also, the degree of muscle wasting was correlated with the progression of carcinogenesis. The late stage of HCC significantly correlated with a higher level of muscle wasting. Interestingly, HCC-induced muscle wasting was not a *kras^G12V^*-oncogene-specific phenotype. We observed in our previous studies that both *Myc*- and *xmrk*-induced liver tumors cause a significant loss of body weight after several months of tumor induction (Li et al., 2012, 2013). Furthermore, we could identify and study the other cachexia phenotypes in our inducible HCC transgenic model.

### Nutrition is not a proper target for treatments of human patients with muscle wasting

Because cachexia patients normally ingest less food, nutritional supplementation and/or appetite stimulation are primary options in conventional treatments on cachexia patients to test whether extra nutrient supply could mitigate the loss of body weight (Ovesen et al., 1993). However, the scientific data on nutrition supplementation for weight-loss cancer patients remains conflicting and controversial (Gullett et al., 2011). Oral supplementation trials showed no effects on gain of body weight in weight-loss patients (Fearon et al., 2003). More serious is the fact that the appearance of substantial muscle wasting could be obscured by the presence of a large fat mass. For example, in a previous study where weight and body composition of 111 human pancreatic cancer patients were investigated, 16.2% patients looked healthier than the overtly weight-loss patients with emaciated body but showed significant sarcopenia (age-related loss of muscle mass and strength) along with being overweight (Tan et al., 2009). In our fish study, we also observed a fattier body in the overfeeding group than in the normal and underfeeding groups but, histologically, the overfeeding group suffered a much more severe muscle wasting owing to accelerated carcinogenesis. In contrast, starvation has been shown to have positive effects on limiting carcinogenesis and improving immunotherapy (Fu and Lee, 2006; Zhong et al., 2016). However, our study indicated one problem of starvation therapy in cancer patients: although underfeeding significantly attenuated carcinogenesis, it could also accelerate muscle wasting due to insufficient nutrient intake. Hence, tumor-derived factors are still the primary cause of muscle-wasting onset and progression (Fearon et al., 2012). Interventions targeting communication between tumor and muscle will be a more effective way to alleviate or even prevent muscle wasting (Johnston et al., 2015).

### Tumor- and overfeeding-induced leptin expression are responsible for HCC-induced muscle wasting

Molecularly, we found a significant upregulation of leptin after *kras^G12V^* induction in the liver. Previously, in a diet-induced obesity model, zebrafish have been found to have an increased body mass index, hypertriglyceridemia and hepatosteatosis with overfeeding, compared with the maintenance control feeding group (Oka et al., 2010). In WT fish, differential feedings had no effect on leptin expression; this is consistent with a previous study in which neither fasting nor long-term satiation feeding affected leptin level in fish (Huising et al., 2006). However, in the *kras^G12V^* model, overfeeding accelerated carcinogenesis and increased leptin expression. Also, fish in the overfeeding groups showed significant increases of fatty liver and higher lipogenic gene expression. In non-alcoholic fatty liver disease (NAFLD) patients, increased severity of NAFLD correlates with higher levels of circulating leptin (Polyzos et al., 2016). In our analyses of human liver disease samples, we found that there were two groups of patients with inflammation: those with either low or high leptin expression. By H&E staining, we found that the high leptin expressing group contained significant lipid droplets, which indicated a higher level of fatty liver. These findings indicated the potential interaction between leptin expression and fatty liver progression. Our finding of high leptin expression in patients with cirrhosis and HCC in human samples was consistent with clinical studies that found that leptin is increased in many liver cirrhosis and HCC patients (Wang and Lin, 2003). Interestingly, the potential driver oncogenes in these human samples could be variable but the leptin level increased in the majority of the samples during liver disease progression, indicating that upregulation of leptin is not specific to a particular oncogene. Hence, leptin signaling is likely universal during liver disease progression from fatty liver to carcinogenesis.

Next, we found high *lepr* expression in skeletal muscle in zebrafish. The leptin receptor is crucial for skeletal muscle function; activation of leptin receptor in human skeletal muscle is associated with muscle hypertrophy in healthy humans (Olmedillas et al., 2010). In our study, *lepr* homozygous mutant zebrafish had a significantly thinner body and smaller muscle fibers after 4 mpf compared to heterozygous mutant and WT fish (Fig. 4E), indicating that *lepr* homozygous mutation affected skeletal muscle development. However, there was no significant difference in feeding, fecundity and mating behavior between homozygous mutant fish and WT (data not shown).

By knockout of the *lepr* gene in zebrafish, we observed significant blockage of muscle wasting, but no effects on liver carcinogenesis. Interestingly, overfeeding alleviated the loss of muscle fibers in *kras^G12V^*/*lepr*^+/−^ fish. Because overfeeding promoted leptin expression, we proposed that leptin was a major signal causing muscle wasting in our *kras^G12V^*-induced HCC model. Hence, we investigated whether signaling components downstream of leptin could provide therapeutic targets to rescue muscle wasting.

### Leptin signaling may be a novel therapeutic target for human patients with muscle wasting

Clinically, myostatin is a master regulator gene to manipulate skeletal muscle mass and function by targeting type-II activing receptor (AcRII) (Gallot et al., 2014). In clinical study, much interest has been devoted to the therapeutic blockage of AcRII signaling. Injection of an ActRIIB antagonist into C26 tumor-implanted mice could significantly block tumor-induced body weight loss and prolong survival (Zhou et al., 2010). Myostatin inhibitor treatment in our *kras^G12V^* fish also significantly alleviated tumor-induced loss of body weight and muscle fibers. Blocking leptin signal activation in skeletal muscle by *lepr* mutation attenuated cancer-associated muscle wasting. Also, we found that chemical inhibition of two main downstream pathways of leptin signal, PI3K/AKT and JAK2/STAT3, showed potential effects on muscle wasting. When we compared the treatment effects of myostatin inhibitor and two chemical inhibitors, we found that, although myostatin-treated fish had the largest muscle-fiber and body weights, the survival rate was lower than the other two treatment groups, although statistically not significant. Based on staining for PCNA+ hepatocytes, we found a significant promotion of tumor cell proliferation by myostatin inhibition. Hence, we concluded that therapeutically targeting communication between tumor and muscle, such as inhibiting leptin signaling, could provide a more efficient treatment than targeting muscle or myostatin.

Interestingly, we found the upregulation of leptin in human patients with various tumor types, further suggesting that leptin is involved in HCC development in humans, as previously reviewed (Wang et al., 2010). There are also numerous reports that leptin is also involved in other cancers. For example, in a cohort of human patients with primary colorectal cancers, leptin expression has been observed in more than half of the patients (Koda et al., 2007); a study on a lung cancer cell line has indicated that leptin promotes progression of lung cancer by inducing proinflammatory cytokines and preventing cell apoptosis (Shen et al., 2009). Hence, leptin signaling plays a stimulating role in the progression of various cancers. As we have identified the stimulating role of leptin on muscle wasting in the zebrafish model, it will be interesting to investigate whether other leptin-expressing tumors also develop muscle wasting after long-term disease progression.

So far, most interest concerning treatments for cancer-induced loss of body weight and muscle fiber have focused on specific nutrition administration, anti-inflammatory drugs, physical exercise and signaling pathways involved in muscle diseases (Aoyagi et al., 2015). Our data provide a new view to treat muscle wasting in leptin-secreting hepatic tumors, and perhaps other leptin-producing tumor types, by inhibiting communication from tumor to muscle, which may provide more effective therapeutic targets.

## MATERIALS AND METHODS

### Zebrafish husbandry

Zebrafish were maintained in compliance with the recommendations in the Guide for the Care and Use of Laboratory Animals of the National Institutes of Health and the protocol was approved by the Institutional Animal Care and Use Committee (IACUC) of the National University of Singapore. Transgenic lines *Tg(fabp10:rtTA2s-M2; TRE2:EGFP-kras^G12V^)* (gz32Tg) in a tetracycline-controlled transcription activation (Tet-On) was used to induce hepatocyte-specific expression of oncogenic *kras^G12V^*, referred to as *kras^G12V^* in the present report (Chew et al., 2014). *lepr* mutant fish were generated using the CRISPR-mediated gene knockdown approach as described previously (Fei et al., 2017).

### Chemical treatments and gross examination

All chemical treatments were performed in 4-month-old adult fish. The chemicals used included doxycycline (dox) (20 μg/ml; D9891; Sigma-Aldrich), human myostatin proform (1 ng/ml; SRP4623P; Sigma-Aldrich), LY294002 (10 μM; L9908; Sigma-Aldrich) and Cucurbitacin I hydrate (10 μM; C4493; Sigma-Aldrich). The dosages were selected based on the highest all-survival concentrations in preliminary experiments. Water with dox and other chemicals was changed twice a week. After chemical treatments, all surviving fish were anesthetized in 0.08% tricaine (E10521; Sigma-Aldrich), immobilized in 3% methylcellulose (M0521; Sigma) and imaged from the left-lateral side with an Olympus microscope.

### Feeding experiments

4-month-old zebrafish were fed once a day in the morning with different doses of artemia for 4 weeks after *kras^G12V^* induction. Based on a previous study (Oka et al., 2010), 5 mg artemia cysts/fish/day of food was used in the maintenance protocol as normal feeding, defined as 100%. Two underfeeding groups with 25% and 50% of normal feeding and two overfeeding groups with 200% and 300% of normal feeding were used in the experiment. Water was collected 2 h post-feeding and the remaining artemia were counted to ensure food intake. Indeed, there was a consistent trend between feeding and food intake (data not shown).

### Histological and immunocytological analyses

All surviving fish were fixed in 4% paraformaldehyde in phosphate-buffered saline (P6748; Sigma-Aldrich) overnight, embedded in paraffin and sectioned at 5-µm thickness using a microtome, followed by various stainings: H&E, IHC, IF, Gomori's trichome and Oil Red O, as we described previously (Yan et al., 2017; Yang et al., 2017a,b). For IHC and IF staining, the primary antibodies included anti-PCNA (FL-261; Santa Cruz Biotechnology; 1:200 dilution), anti-Hnf4a (SC-6556; Santa Cruz Biotechnology; 1:200 dilution) and anti-Leptin (Ab1673; Merck; 1:100 dilution). Anti-rabbit or anti-goat secondary antibodies were purchased from Thermo Fisher Scientific.

### RNA extraction and RT-qPCR

Total RNA was extracted using the RNeasy Mini Kit (Qiagen), and 5 ng RNA was used as a template to synthesize and amplify cDNA using the QuantiTect Whole Transcriptome Kit (Qiagen). RT-qPCR was conducted with LightCycler 480 SYBR Green I Master (Roche Diagnostics). Genes of interest were amplified for 40 cycles (95°C, 20 s; 65°C, 15 s; 72°C, 30 s). The primers used and their sequences are shown in Table S1.

### Human patient samples

Human liver disease progression tissue microarray slides were purchased from Biomax, Inc. (LV8011a). Patients were classified into four groups: normal liver, inflammation, cirrhosis and HCC. Histopathology of all patients was diagnosed and provided by the company. Patient sample slides were subjected to IHC staining for human leptin.

### Statistical analyses

A two-tailed unpaired Student's *t*-test was performed using GraphPad Prism version 7.00 for statistical significance between two groups. Statistical data are presented as means±s.e.m. Significance is indicated with an asterisk if *P*<0.05.

## Supplementary Material

Supplementary information
