# Supplementary material for: Leptin induces muscle wasting in a zebrafish kras-driven hepatocellular carcinoma (HCC) model
Source: Dis Model Mech. 2019 Feb 27;12(2):dmm038240. doi: 10.1242/dmm.038240 (PMC6398506; doi:10.1242/dmm.038240)
Supplement: Supplementary information [file dmm-12-038240-s1.pdf]

Table S1. PCR primer sequences

Gene name	Forward sequence	Reverse sequence
β-actin	CTCTGGGTCACCGCTTCTTT	CAGATGCTCACGAAACCCCT
crebp1	TGAAGATTGGCGATCGAGGG	ATTTTCGCCTTGTCCCGACT
pparg	CACTCTCCGCTGATATGGTGG	GTAGATGGGCTCGTGTGTCC
srebp1	CTCTGGGTCACCGCTTCTTT	CAGATGCTCACGAAACCCCT
lepa	GAGATTCCCGCTGACAAACC	CAGTCCATGCCTTCCGGTAA
lepr	CGCGCTTGTATCCTCATGTT	GAGAGAGAGATTTTGTGCGA

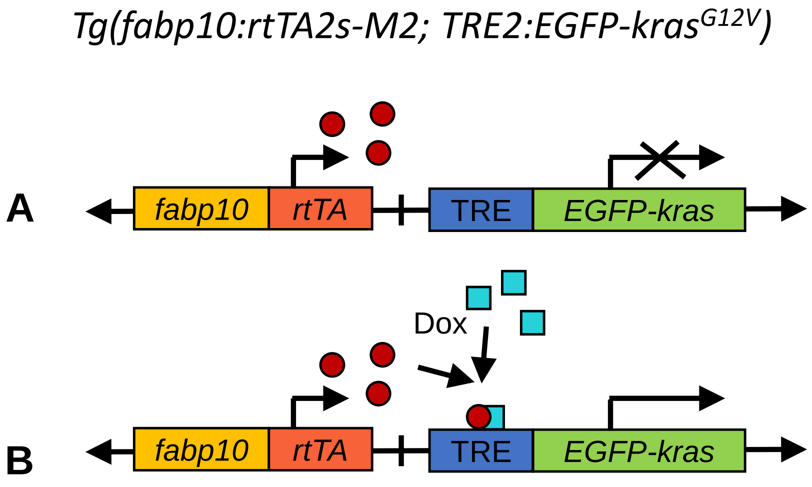

Fig. S1. Schematic illustration of the Dox-inducible system used in kras+ transgenic zebrafish *Tg(fabp10:rtTA2s-M2; TRE2:EGFP-kras^{G12V})*. *rtTA* (reverse tetracycline transactivator) mRNA is transcribed under the liver-specific *fabp10* promoter and *EGFP-kras* is controlled by TRE (tetracycline responsive element). (A) in the absence of doxycycline (Dox), an analog of tetracycline, rtTA could not bind to TRE and thus *EGFP-kras* is not transcribed. (B) When Dox is present, it binds to rtTA to cause the latter to bind to TRE and to transcribe the *EGFP-kras* oncogene.

Disease Models & Mechanisms • Supplementary information

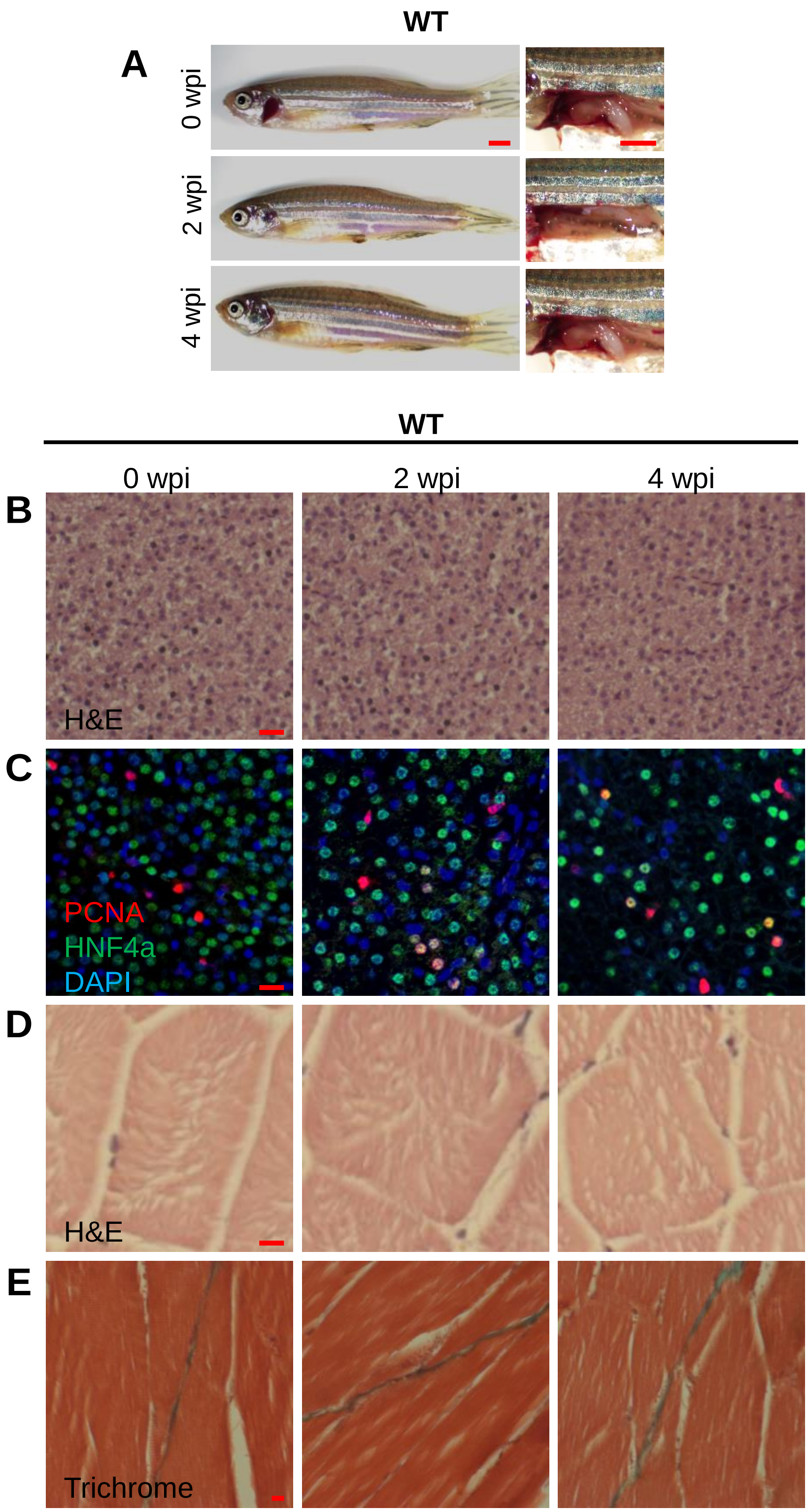

Fig. S2. Images of WT control zebrafish to supplement Fig. 1. 4-month-old male adult WT zebrafish were treated with dox for 4 weeks and sampled at 0 wpi, 2 wpi and 4 wpi. (A) Gross appearance and liver morphology (left lateral view). (B) H&E staining of liver sections of WT fish. (C) IF staining of PCNA (red), Hnf4a (green) and DAPI (blue) in liver sections of WT fish. (D) H&E staining of muscle sections of WT fish. (E) Gomori's trichrome staining of muscle sections of WT fish. Scale bar: 2.5 mm in (A); 10 μm in (B-E).

Disease Models & Mechanisms • Supplementary information

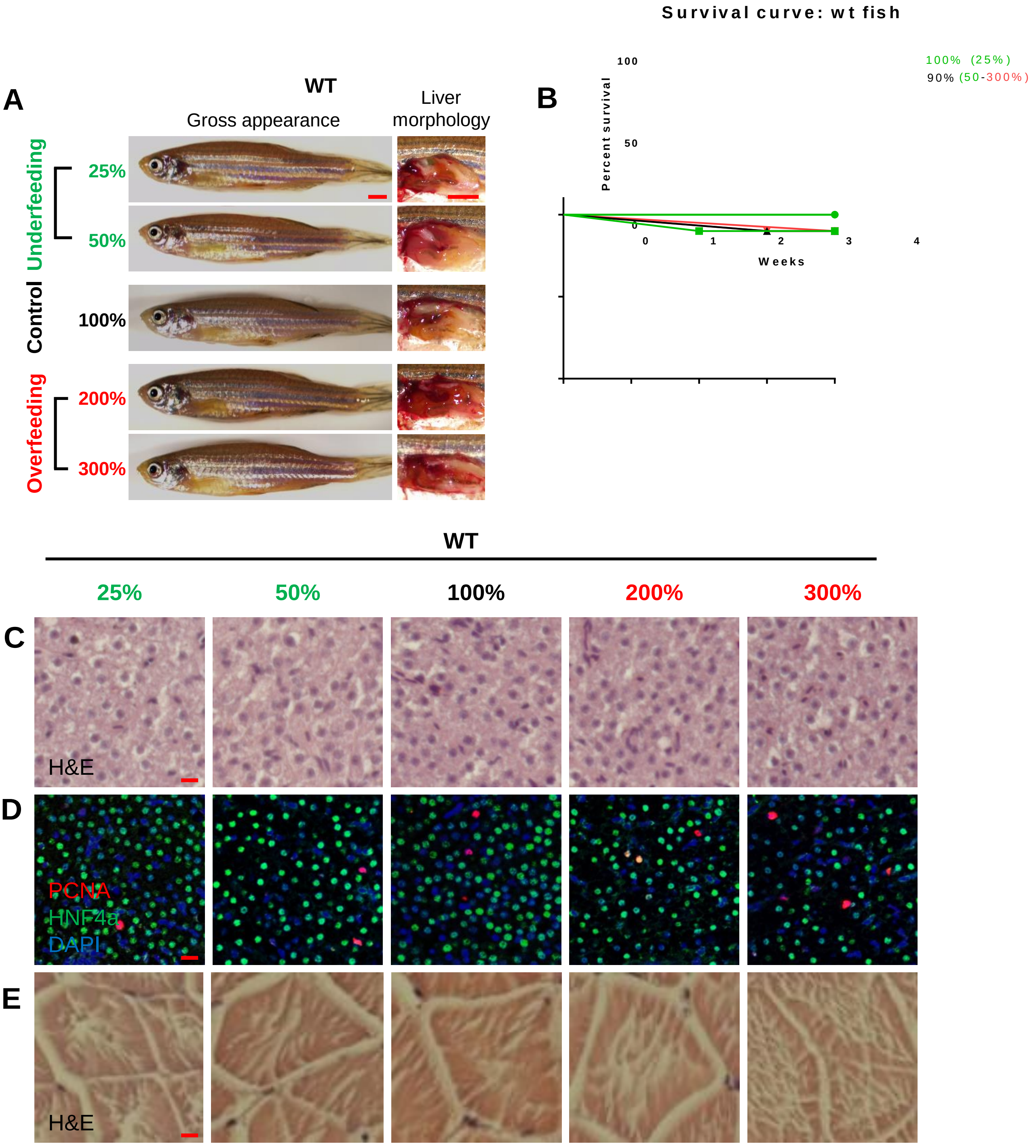

Fig. S3. Images of WT control zebrafish to supplement Fig. 2. 4-month-old male adult WT zebrafish were treated with dox for 4 weeks and fed with different level of artemia as described before. 10 fish were used in each group. (A) Gross appearance and liver morphology (left). (B) Survival curves of WT fish fed with different level of food. (C) H&E staining of liver sections of WT fish. (D) IF staining of PCNA (red), Hnf4a (green) and DAPI (blue) in liver sections of WT fish. (E) H&E staining of muscle sections of WT fish. Scale bar: 2.5 mm in (A); 10 μm in (C-E).

Disease Models & Mechanisms • Supplementary information

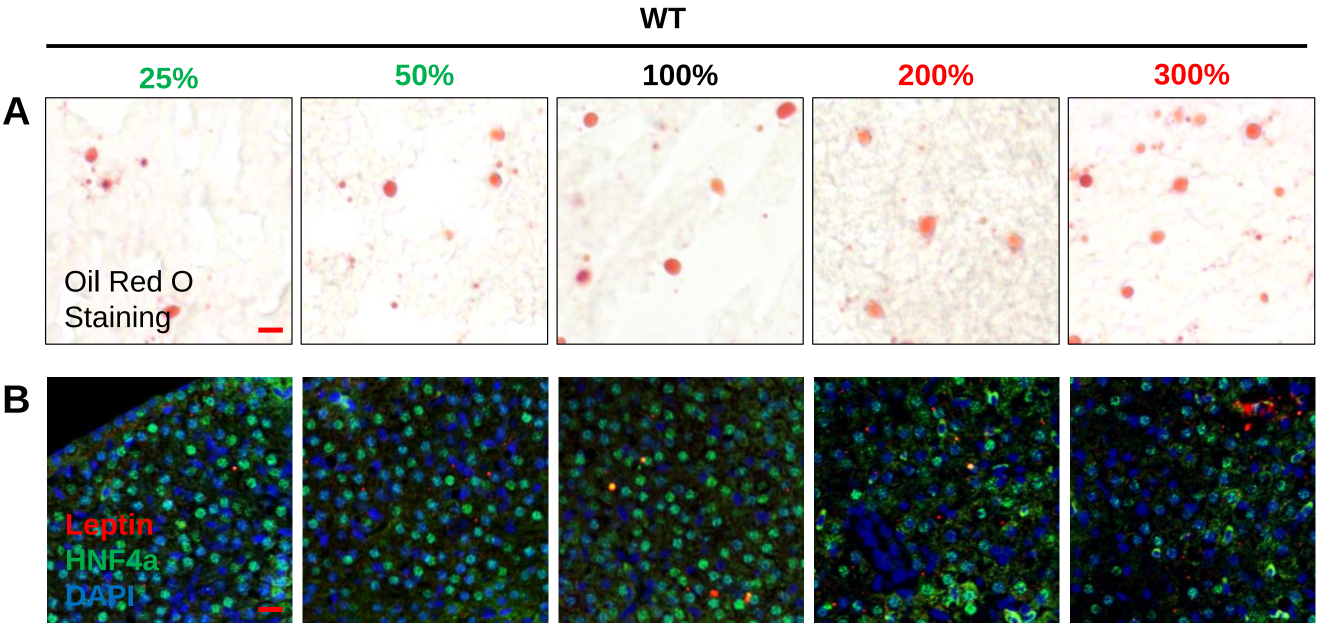

Fig. S4. Images of WT control zebrafish to supplement Fig. 3A and 3C. 4-month-old adult WT male zebrafish were treated with dox for 4 weeks and fed with different level of artemia. (A) Oil Red O Stain of liver sections of WT fish. (B) IF staining of leptin (red), Hnf4a (green) and DAPI (blue) in liver sections of WT fish. Scale bar: 10 µm.

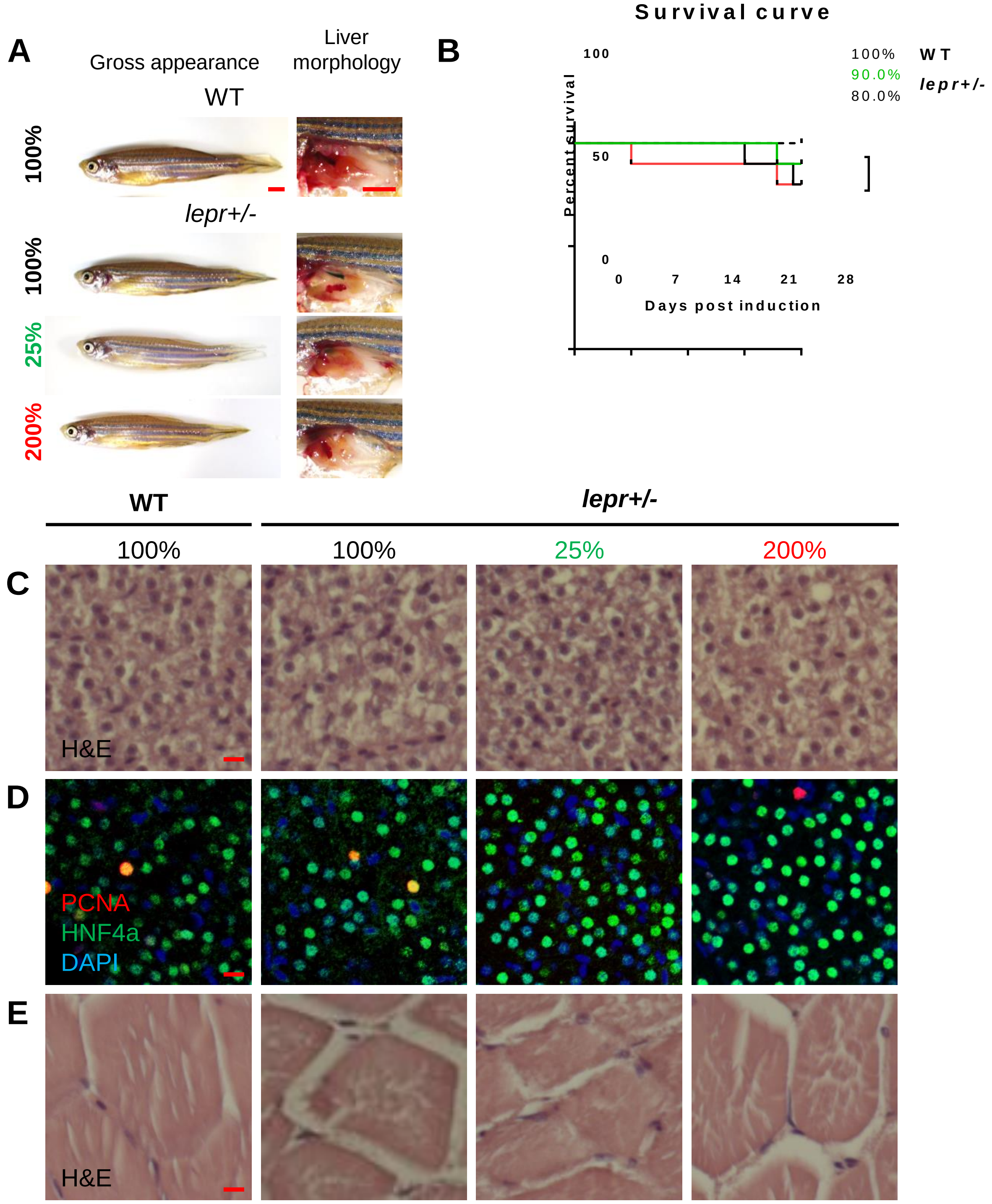

Fig. S5. Images of WT control zebrafish to supplement Fig. 5. WT fish under normal feeding and heterozygous mutant fish under 25%, 100% and 200% feeding were treated with dox for 4 weeks. 10 fish were used in each group. (A) Gross appearance and liver morphology (left). (B) Survival curves. (C) H&E staining of liver sections. (D) IF staining of PCNA (red), Hnf4a (green) and DAPI (blue). (E) H&E staining of muscle sections. Scale bar: 2.5 mm in (A); 10 μm in (C-E).

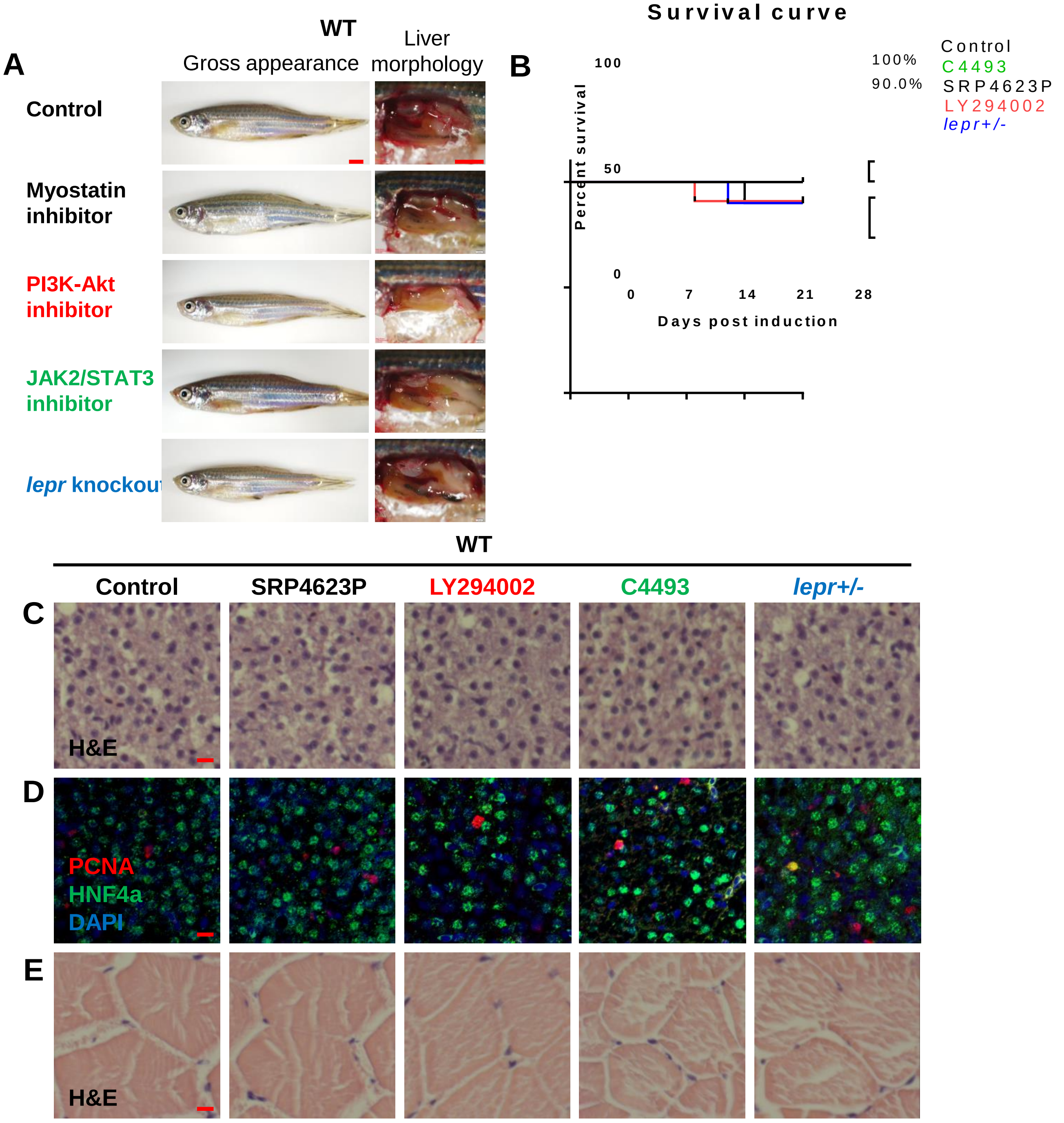

Fig. S6. Images of WT control zebrafish to supplement Fig.6. 4-month-old male adult WT zebrafish were treated with dox for 4 weeks with SRP4623, LY294002 and C4493, respectively. Heterogeneous mutant fish were treated with dox for 4 weeks. 10 fish were used in each group. (A) Gross appearance and liver morphology (left). (B) Survival curves. (C) H&E staining of liver sections of WT fish. (D) IF staining of PCNA (red), Hnf4a (green) and DAPI (blue) of WT fish. (E) H&E staining of muscle sections of WT fish. Scale bar: 2.5 mm in (A); 10 μm in (C-E).

Disease Models & Mechanisms • Supplementary information
